# Realist Collaborative Evaluation of a Work Disability Prevention Program for Breast Cancer Survivors: Protocol for a RECOVA-FASTRACS Realist Evaluation

**DOI:** 10.2196/86608

**Published:** 2026-07-28

**Authors:** Apolline Blazer, Sabrina Rouat, Laure Guittard, Pauline Drouin, Julien Péron, Béatrice Fervers, Julien Carretier, Guillaume Broc, Laurent Letrilliart, Jean-Baptiste Fassier, Marion Lamort-Bouché

**Affiliations:** 1 INSERM U1290, University Claude Bernard Lyon 1, Research on Healthcare Performance (RESHAPE), 8 Avenue Rockefeller, Lyon, 69008, France, 33 0770979715; 2 Université Lyon 2, Groupe de Recherche en Psychologie Sociale (GRePS), Lyon, France; 3 Pôle Santé Publique, Service Recherche et Epidémiologie Cliniques, Hospices Civils de Lyon, Lyon, France; 4 Institut de Cancérologie, Service d'Oncologie Médicale, Hospices Civils de Lyon, Lyon, France; 5 Département Cancer et Environnement, Centre Léon Bérard, Lyon, France; 6 INSERM U1296, Radiations: Defense, Heath & Environnement, Lyon, France; 7 UR4129, Université Claude Bernard Lyon 1, Parcours Santé Systémique (P2S), Lyon, France; 8 Centre National des Soins Palliatifs et de la Fin de Vie, Paris, France; 9 EA 4556, Université Paul-Valéry Montpellier 3, Dynamique des Capacités Humaines et des Conduites de Santé (EPSYLON), Montpellier, France; 10 Université Claude Bernard Lyon 1, Collège Universitaire de Médecine Générale (CUMG), Lyon, France; 11 UMR T9405, Université Claude Bernard Lyon1, Unité Mixte de Recherche Epidémiologique et de Surveillance Transport Travail Environnement, Lyon, France

**Keywords:** program evaluation, evaluation studies as topic, realist evaluation, cancer survivors, breast neoplasms, return to work, primary health care

## Abstract

**Background:**

Women with breast cancer face many barriers to returning to work (RTW) after their treatment. The Facilitating and Sustaining the Return to Work After Breast Cancer (FASTRACS) intervention aims to facilitate and sustain functional RTW.

**Objective:**

The main objective of the RECOVA-FASTRACS (Realist Collaborative Evaluation of a Work Disability Prevention Program for Breast Cancer Survivors) study is to evaluate the processes using a realist approach that analyzes what works, how, for whom, and under what circumstances.

**Methods:**

The RECOVA-FASTRACS study uses a mixed methods design to assess the implementation, context, and impact mechanisms of the FASTRACS intervention. The qualitative analysis will include 2 main components: a trajectory analysis and a focus group assessment. The trajectory analysis will examine the experiences of women who participated in the intervention and key individuals involved in their RTW process. We will use semistructured interviews according to the multiple-case study method. Additionally, to explore organizational and professional practices, focus groups will be conducted with professionals who deliver the intervention. To analyze the trajectories, embedded and iterative integration will combine the qualitative findings with relevant quantitative data from the FASTRACS randomized controlled trial for 5 domains: personal situation, professional situation, RTW, care pathway, intervention tool use, and perceived usefulness.

**Results:**

The RECOVA-FASTRACS study received funding in 2022. Recruitment and qualitative data collection began in month 6. Final analyses are expected to be completed by the end of 2026, with dissemination of the main findings anticipated in late 2027.

**Conclusions:**

Our mixed methods realist evaluation will provide a detailed analysis of the intervention processes, helping to identify impact mechanisms within specific contexts. This approach is meant to ensure a more informed and realist deployment of the intervention by professionals following the study.

## Introduction

### Background

In Europe, 5.4 million women were living with breast cancer in 2020 [1]. In France, more than 61,000 new cases are diagnosed every year, half of them in women of working age [2]. More than 254,000 patients diagnosed with breast cancer in the past 5 years are still alive [3]. While the incidence of breast cancer continues to rise, especially among women under the age of 50 years [4], the retirement age continues to increase. Consequently, returning to work (RTW) after breast cancer is essential for women wishing to resume a professional life [5], ensuring their financial independence and social participation while reducing the social costs associated with the disease [3].

However, research has identified several obstacles that impede women’s RTW [6,7]. These obstacles are associated with the consequences of the disease and its treatment (fatigue, pain, and cognitive problems) and the age of the patients [8,9]. Interprofessional collaboration could effectively support these patients in the postcancer phase, but barriers continue to hinder collaboration between breast cancer specialists, general practitioners, and occupational physicians [10]. In the work environment, professional demands may not be compatible with the patients’ situations. Moreover, a lack of knowledge about the disease, along with insufficient support from superiors and colleagues, may complicate RTW [11,12]. Sociodemographic inequalities also influence RTW, with a less favorable occupational prognosis in older and less-qualified people [13,14].

The importance of RTW has been formalized in France’s 10-year cancer strategy for 2021 to 2030 [15]. Employers are implementing measures to support employee reintegration, including the “Cancer and Employment” charter established by the French National Cancer Institute. The charter seeks to provide additional assistance for individuals undergoing or recovering from cancer treatment [16].

Multidisciplinary interventions appear to increase the number of people who RTW [17]. These interventions are filling the gaps left by overly medicalized interventions, which have been criticized for their weak theoretical basis. Such a basis has proved insufficient for analyzing the causes (problem theory) of obstacles and for proposing solutions (action theory) [18,19].

The Facilitating and Sustain the Return to Work After Breast Cancer (FASTRACS) research program [20] was developed using the intervention mapping framework [21]. It addresses the need for multidisciplinary interventions [22] by engaging various RTW stakeholders early in the cancer care process. The intervention consists of an RTW pathway for breast cancer survivors (BCSs) that connects the hospital, the primary care team, and the workplace environment, starting when active treatments have concluded [20]. This pathway is structured around 4 tasks for supporting BCSs:

- Regularly assess their needs and find relevant information using the patient guide specifically created for this purpose [23].
- Schedule an appointment dedicated to RTW anticipation with their general practitioner.
- Schedule an appointment with their occupational physician.
- Maintain or reestablish contact with their workplace environment.

With these tasks completed, BCSs can anticipate the potential professional challenges associated with RTW and optimize their comeback.

However, the process of change cannot rely on women alone and must target the behaviors of other stakeholders as well (eg, general practitioners, occupational physicians, managers, and so on), in line with the recommendations on complex interventions [24]. Four tools have thus been developed to facilitate the process, mobilizing the key actors in the RTW process:

- A *patient guide* structured in 3 sections: an informative section detailing 4 key tasks to prepare for RTW, a personal development section to support this process and prevent difficulties, and a resource directory listing relevant services and tools.
- A *checklist for the general practitioner* to standardize the assessment of needs, the design of post–breast cancer care, and the RTW plan.
- A *checklist for the occupational physician* to standardize the assessment of aptitude and the adaptation of the workstation to prepare for the RTW among BCSs.
- An *employer’s guide* to help local managers, human resources, employers, and occupational physicians understand the challenges of employee reintegration. It provides guidance on ensuring optimal working conditions, adapting job roles, facilitating internal job transitions when needed, and offering support for external career opportunities if necessary.

A randomized controlled trial (RCT) is currently underway (FASTRACS-RCT, NCT04846972) in 14 investigation centers. The trial will evaluate the effects of the FASTRACS intervention on the sustainable RTW of BCSs 12 months after the end of active treatment [25].

RTW interventions are considered complex interventions due to the diverse influencing factors, the multiple actors involved, and the variety of implementation contexts [26]. Such interventions have a heightened risk of failure in terms of delivery and sustainability due to the dynamic interactions among their components. To better anticipate these challenges, the Medical Research Council recommends evaluating these interventions to gain a contextualized understanding of how they induce changes while analyzing the factors that facilitate or hinder their delivery [26].

Therefore, in addition to conducting a quantitative evaluation of the effects, it is necessary to evaluate the intervention processes [27]. This process evaluation serves 3 key purposes: it provides a dynamic analysis of the intervention’s implementation, examines the mechanisms through which it generates impact, and identifies the contextual factors that influence these mechanisms.

### Objectives

The main objective of the RECOVA-FASTRACS (Realist Collaborative Evaluation of a Work Disability Prevention Program for Breast Cancer Survivors) study is to conduct a process evaluation of the FASTRACS intervention by examining its implementation, context, and impact mechanisms. More specifically, this ancillary study will assess what has been implemented and how, while identifying barriers and facilitators influencing the implementation of the intervention components. In parallel, the context analysis will explore how specific contextual factors affect the intervention’s implementation and outcomes. Finally, the study of impact mechanisms will examine how the intervention produces the expected effects and any unintended consequences that may arise.

The secondary objective is to refine the initial propositions of intervention theory by confirming or revising them through the elaboration of middle-range theories.

## Methods

The reporting of the study protocol follows the RAMESES II (Realist And Meta-narrative Evidence Syntheses—Evolving Standards) reporting standards for realist evaluation and the GRAMMS (Good Reporting of a Mixed Methods Study) guidelines (Checklist 1) [28,29].

### Realist Evaluation as a Process Evaluation Approach

Process evaluation can be carried out using various methodological approaches. Among these, realist evaluation [30,31] stands out for its systematic approach to the interactions between intervention and context. Stemming from the theory-based evaluation movement [32], realist evaluation aims to answer the question of “what works, how, why, for whom, and in what circumstances” [30] by identifying context-mechanism-outcome (CMO) configurations. By analyzing these configurations together, researchers can define “demi-regularities” and develop middle-range theories that explain the variations in observed effects [33]. By comparing these theories with the intervention’s initial assumptions in the theory of intervention, the researcher can refine the intervention theory. Applying this approach to the FASTRACS intervention makes it possible to dynamically analyze its implementation and impact mechanisms, supporting its future adaptation and scalability in real-life settings.

### Study Design

RECOVA-FASTRACS is a realist process evaluation conducted as a mixed methods ancillary study to FASTRACS-RCT to refine the initial program theory (publication forthcoming and Broc et al [23]). In this study, the mixed methods approach refers to the integration of qualitative and quantitative data within the overarching realist evaluation framework. It uses a convergent design within a case-study framework [34]. The study integrates theory-testing interviews through a multiple-case study with quantitative data from FASTRACS-RCT using an embedded approach to triangulate findings.

The qualitative research will be divided into 2 parts:

- Substudy A: a study of the trajectories of the BCSs who have completed follow-up in the FASTRACS-RCT intervention group (RCT-I). This study aims to analyze the RTW pathway of these participants, examining the role of the intervention in this process.
- Substudy B: a study focusing on the professionals involved in the FASTRACS-intervention delivery.

In substudy A, we will analyze the trajectories of RCT-I, focusing on participants’ experiences of the intervention through their own narratives. These results will then be triangulated with those of the people who contributed to their RTW pathway. Data will be collected through semistructured interviews scheduled at the end of the RCT-I follow-up in FASTRACS-RCT to prevent contamination risks.

Simultaneously, substudy B will explore how professionals deliver the intervention. We will conduct focus groups to gather multiple perspectives, allowing for interaction on the topics discussed. This approach will facilitate the identification of organizational dynamics and professional practices specific to each professional [35].

In quantitative research (substudy C), we will analyze data from the RCT questionnaires completed by RCT-I.

Only participants from the intervention arm will be included in the qualitative trajectory analysis, as the objective of the realist evaluation is to explore the implementation processes, mechanisms, and contextual conditions associated with exposure to the FASTRACS intervention. The study does not aim to qualitatively compare intervention and usual-care trajectories. However, experiences related to usual care, RTW barriers, and the ordinary experience of living with breast cancer and RTW have already been extensively explored during the needs assessment phase of the intervention mapping process [20]. In addition, the profession-specific focus groups conducted with professionals involved in RTW support will contribute to documenting routine professional practices and usual-care conditions experienced by women participating in the FASTRACS-RCT. Quantitative data from the FASTRACS-RCT, including participants from both intervention and usual-care groups, will further support contextualization and interpretation of the intervention-specific mechanisms identified through the realist analysis.

**Figure 1.**
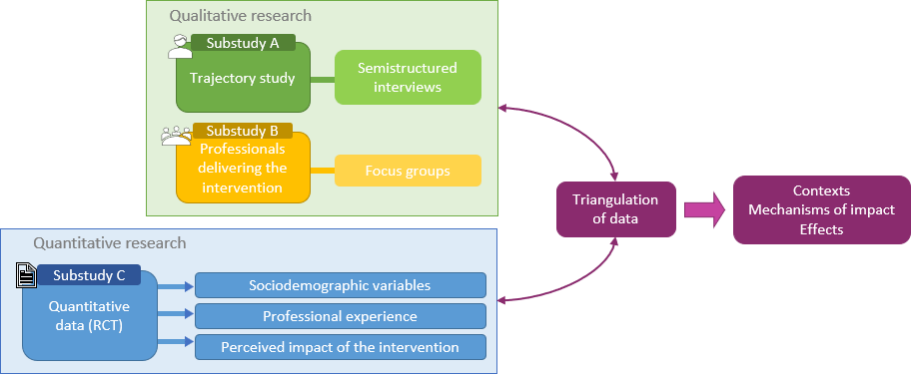
RECOVA-FASTRACS (Realist Collaborative Evaluation of a Work Disability Prevention Program for Breast Cancer Survivors) study design. RCT: randomized controlled trial.

Triangulation of qualitative findings with quantitative data will allow the formulation of hypotheses on the implementation, underlying contexts, mechanisms, and effects that the intervention might produce on the RCT-I. Figure 1 illustrates the design of the study.

### Recruitment Process and Sampling Strategies of Studies

#### Substudy A: Trajectory Analysis

To explore the various experiences of those participating in the intervention, RECOVA-BCS will constitute a sample of typical cases [36] among RCT-I as “the most representative of the phenomenon being explored.” It will refer to the most frequently observed profiles identified during sampling and early analysis, representing recurrent RTW trajectories within the dataset. This RECOVA-BCS sampling will then be conducted to construct a theoretical sample that maximizes variation based on factors, such as age, occupation, and place of residence.

RECOVA-BCS selected for the study will be asked for permission to interview individuals who helped them in their RTW pathway. This population is divided into 2 groups:

- Program theory actors (PTAs): general practitioners, occupational physicians, and workplace stakeholders, who have been preidentified as key actors in the RTW pathway and are directly involved in the intervention through specific tools (such as checklists and employer’s guide).
- RTW supporters: individuals identified by RECOVA-BCS participants as having provided significant support during the post–heavy treatment period and RTW. They can include health professionals (eg, physiotherapists and nurses), workplace individuals (eg, colleagues), and up to 3 relatives.

Due to the iterative nature of qualitative methodology, sampling of RECOVA-BCS, PTAs, and RTW supporters will be progressively guided by the initial program theory of the FASTRACS intervention (publication forthcoming), translated into intervention-context-actor-mechanism-outcome (ICAMO) configurations. Sampling will aim to ensure sufficient variation to test and refine this theory across different contexts as the analysis evolves. Data collection will be carried out until saturation is reached [37], defined as the point at which ICAMO configurations are sufficiently stable and no further refinement of the program theory emerges from additional cases, including situations where expected mechanisms are not activated in specific contexts.

We anticipate including approximately 20 RECOVA-BCS in individual interviews over time to gather sufficient information on how the intervention is experienced by a large diversity of profiles. This sample size aligns with qualitative and realist-informed studies. For each RECOVA-BCS, interviews will also be conducted with up to 4 individuals involved in their RTW support, including at least one PTA, allowing for the triangulation of perspectives across key actors involved in the RTW process. We expect to identify between 10 and 15 theoretical pathways (ie, the patient and 1 PTA) to maximize the diversity of RTW trajectories. However, this is an iterative process, and the reality on the ground may lead us to revise our initial sampling.

At the end of the FASTRACS-RCT, deviant and crucial cases [36] may be identified and included to deepen the understanding of the intervention and its mechanisms. These cases will be identified through the quantitative component of the study, based on results that appear atypical or particularly informative in relation to the questionnaire data. This may include extreme values, as well as trajectory patterns identified through cluster-based analyses distinguishing groups with faster versus more delayed RTW processes.

#### Substudy B: Professionals Delivering the Intervention

To analyze how the intervention is implemented, professionals delivering the intervention will be identified at every stage of the FASTRACS intervention, including professionals such as the RCT participants (oncologists and clinical research associates), general practitioners, occupational health teams (occupational physicians or occupational nurses), and staff from the Regional Center for Occupational and Environmental Pathologies. Sampling will be theoretical and aims to maximize variation in characteristics, such as age, gender, professional role, geographical area, and the number of BCSs included in the RCT they supported.

The categories of professionals are as follows:

- Oncologists: physicians who have enrolled BCSs in the FASTRACS-RCT.
- Clinical research associates: those tasked with delivering the intervention tools to RCT-I.
- General practitioners: physicians providing care to RCT-I, identified through the RCT database.
- Occupational health teams: teams identified through the RCT database, including those from the Regional Center for Occupational and Environmental Pathologies.

Approximately 10 profession-specific focus groups are expected to be conducted to capture a wide range of perspectives on the implementation of the intervention, particularly regarding contextual conditions and intervention delivery across settings and professional categories. When group constitution proves difficult due to recruitment or scheduling constraints, semistructured individual interviews will be conducted instead.

The number of focus groups will depend on data saturation [37] under a realist perspective defined here as the point at which no new ICAMO configurations related to implementation processes, contextual influences, or professional responses emerge, and when additional data no longer contribute to the refinement of the program theory.

#### Substudy C: Quantitative Data

In this mixed methods study, quantitative data will be drawn from the FASTRACS-RCT questionnaires that were administered to RCT-I. The characteristics of this study population are detailed in the FASTRACS-RCT protocol [25].

### Data Collection

#### Substudy A: Trajectory Analysis

The trajectory analysis will examine the experience of the FASTRACS intervention from the perspective of RECOVA-BCS and the individuals who supported them. To do so, it will explore the extent to which the intervention influenced their RTW pathway by studying its implementation, impact mechanisms, and the effects it might have produced while considering the contexts in which the intervention took place.

Each identified RECOVA-BCS, RTW supporter, and PTA will be interviewed individually. Each interview will take place at the location and using the modality chosen by the participant (face-to-face, videoconference, or telephone).

Five interview guides (Multimedia Appendix 1) were developed using a hypothetico-deductive approach, based on the methodology of the explanatory interview [38] and guided by the realist interviewing process of conceptual focusing [39]. The guides were developed from the initial FASTRACS program theory (publication forthcoming and Broc et al [23]) and its hypothesized intervention effects but were intentionally designed around open-ended and narrative questions to minimize the induction of expected responses and limit social desirability bias. This approach aims to leave sufficient space for participants to describe their lived experiences and trajectories in their own terms, while still allowing exploration of mechanisms, contextual conditions, and perceived effects related to the intervention.

These guides have been designed to meet the objectives of the intervention and are adapted to the different categories of interviewees. The open-ended questions in the guides are both narrative (to explore personal experiences) and structural (to identify the key stages). This structure is intended to capture how each participant experienced and perceived the intervention. The guides were reviewed and adjusted after the first interviews and, in line with realist evaluation principles, may continue to evolve iteratively throughout the study to further explore emerging ICAMO configurations and unresolved analytical questions. The themes of the interview guides are shown in Table 1.

**Table 1.** Interview themes by participant type.

Participant	Interview guide themes
RECOVA-BCS^a^	The decision to RTW^b^ and the associated experiences Relationship to work Use and appreciation of the patient guide Support received from PTA^c^ Emotional and social support from RTW supporters Process of reestablishing contact and links with the company
RTW supporters	Medical opinion on the situation of the RECOVA-BCS (if health professional) Emotional and social support given and received Knowledge of the relationship between RECOVA-BCS and work Use and appreciation of the patient guide
General practitioner	Consultations with the patient RTW support Side effects and RTW Emotional and social support from those around the RECOVA-BCS Use and appreciation of the checklist Knowledge and use of the patient guide Professional coordination and its perceived effects
Occupational physician	The role of RECOVA-BCS in the company The RTW career path of RECOVA-BCS and the factors that hinder or facilitate it Knowledge and use of the employer’s guide Use and appreciation of the checklist Knowledge and use of the patient guide
Workplace actors	Workplace description RTW of the RECOVA-BCS Use and assessment of the employer’s guide Actions taken and resources mobilized Development of new managerial skills

a RECOVA-BCS: sample of typical cases among breast cancer survivors who completed follow-up in the FASTRACS-RCT (FAcilitating and SusTain the Return to work After breast Cancer randomized controlled trial) intervention group.

b RTW: return to work.

c PTA: program theory actor.

#### Substudy B: Professionals Delivering the Intervention

In this substudy, the experiences of professionals delivering the FASTRACS intervention will be explored to analyze its implementation. These focus groups will be organized in parallel with the semistructured interviews of substudy A and after their completion, to allow triangulation and clarification of the information collected. Each focus group will last between 1.5 and 2 hours. They will be conducted with the various categories of stakeholders involved (oncologists, clinical research associates, general practitioners, and occupational health teams).

A focus group guide was designed for each category of participants following a realist evaluation approach [40]. This guide is structured around themes relating to the experience of the intervention, the obstacles and facilitators encountered during its implementation, its integration into professional practice, and its appropriation by those delivering the intervention. In line with conceptual focusing principles, the guides may be refined iteratively throughout the study to further investigate emerging ICAMO configurations. The main themes of the guides are shown in Textbox 1.

Textbox 1. **Interviewer questions guide themes for professionals delivering the intervention.**

**Focus group guide themes**

· How they got involved?

· Pathway of the RCT-I (breast cancer survivors who completed follow-up in the FASTRACS-RCT intervention group)

· Use and appreciation of the intervention tools

· Experience of the research in the context of their professional practice

To minimize hierarchical or professional power imbalances, profession-specific focus groups will be organized separately for each stakeholder category and will follow the recommendations of our participatory committee (comprising representatives of doctors and business representatives). The facilitators will actively encourage balanced participation and constructive discussion to ensure that all participants can freely express their views and experiences.

#### Substudy C: Quantitative Data

To complement the qualitative data gathered through semistructured interviews, we will extract relevant items related to intervention processes from the questionnaires completed as part of the FASTRACS-RCT [25], which are securely stored in the Ennov database. The full list of questions can be found in Multimedia Appendix 2. These questions are selected for their relevance to the analysis of key aspects of the intervention and will focus on 5 domains: personal situation, professional situation, RTW, care pathway, and the use and assessment of intervention tools.

Quantitative data management is carried out in the context of the FASTRACS-RCT by the project team (Selma Baka, Aimline Soulier, and PD) and is, therefore, described elsewhere [25]. This team is affiliated with *Hospices Civils de Lyon* and is, therefore, independent of the funders.

We will first jointly analyze the qualitative and quantitative data from the RECOVA-BCSs and further compare their trajectories with those of the broader FASTRACS-BCS population using embedded integration.

### Data Analysis

The realist analysis will be carried out in an extended context-mechanism-outcome configuration: ICAMO [41]. The intervention is defined as the resources and components delivered by the program (eg, patient guide, checklists, and coordination procedures). Context refers to external and individual conditions shaping the activation of mechanisms, including organizational settings, health care pathways, workplace environments, interpersonal relationships, and personal circumstances embedded within different ecological levels of the patient’s situation [42,43]. Actors are individuals or groups whose behavior or reasoning is targeted by the intervention [44]. Mechanisms are defined as changes in reasoning and reactions that bring about change [45]. Outcomes refer to the observable effects generated by these mechanism-context interactions [46].

To avoid any confusion between intervention components and mechanisms, a structured decision rule will be applied during analysis: (1) if a statement describes “what the program provides,” it will be coded as intervention; (2) if it describes “what is happening in the environment,” it will be coded as context; (3) if it describes “what actors think, decide, or do in response to resources within a context,” it will be coded as mechanism.

For example, the patient guide is an intervention component; being provided with the guide is the intervention exposure; feeling reassured or legitimized to discuss RTW with health care providers after reading it is a mechanism; and organizational support from an employer facilitating reduced working hours is a contextual condition influencing whether this mechanism is activated.

### Qualitative Analysis

A 2-phase thematic analysis of qualitative interviews:

- Deductive coding processes will be applied using the initial program theory of the FASTRACS intervention (publication forthcoming), which has been previously translated into ICAMO configurations. This step will allow for the identification of key actors, intervention components, and the mapping of RTW pathways in relation to the initial theoretical propositions.
- Inductive coding analysis will identify the ICAMO configurations underlying the facilitators and barriers to the intervention’s effectiveness [31].

To enhance rigor, at least 2 researchers will independently analyze the transcripts before engaging in counterfactual reasoning and judgmental rationality, comparing and refining the ICAMO configurations. MAXQDA 24 software (MAXQDA) will be used to integrate and compare the entire corpus.

### Quantitative Data Integration

Quantitative data from FASTRACS-RCT surveys (collected at baseline, 4, 8, and 12 mo) will be analyzed following the prespecified statistical analysis plan [25]. In addition, subgroup analyses (eg, by socioprofessional category, age, center, and company size) will be used to test the hypotheses developed during the interviews.

### Embedded and Iterative Integration

Qualitative and quantitative data will be analyzed in parallel and will be integrated sequentially [47]. For each RECOVA-BCS, qualitative data will be directly embedded into their corresponding quantitative dataset (Multimedia Appendix 2), which includes selected variables from the RCT, such as sociodemographic characteristics (age, occupation, company size, geographical area), RTW outcomes, care pathway indicators, and the use and perceived usefulness of intervention tools. This embedding will allow within-case comparisons and exploration of how qualitative findings relate to quantitative patterns.

Where findings converge, they will be used to strengthen the interpretation of emerging ICAMO configurations. In cases of divergence between qualitative and quantitative data, these discrepancies will be systematically documented and explored as potentially informative for refining the program theory (eg, revealing contextual contingencies or alternative mechanisms).

Integration will be conducted at the analysis level through the use of structured matrices and joint displays [47], which will allow visual and analytical comparison of qualitative and quantitative findings across cases. These tools will also be used iteratively to identify areas of convergence and divergence between datasets. Importantly, this ongoing integration will inform subsequent data collection by highlighting aspects that appear underexplored in qualitative interviews but are emerging from quantitative patterns, thereby guiding the development of additional or more focused interviews to further investigate these discrepancies.

This embedded analysis will allow for constant iteration between empirical observations and the initial program theory, progressively defining middle-range theories that hypothesize for whom, how, and under what conditions the intervention works.

**Figure 2.**
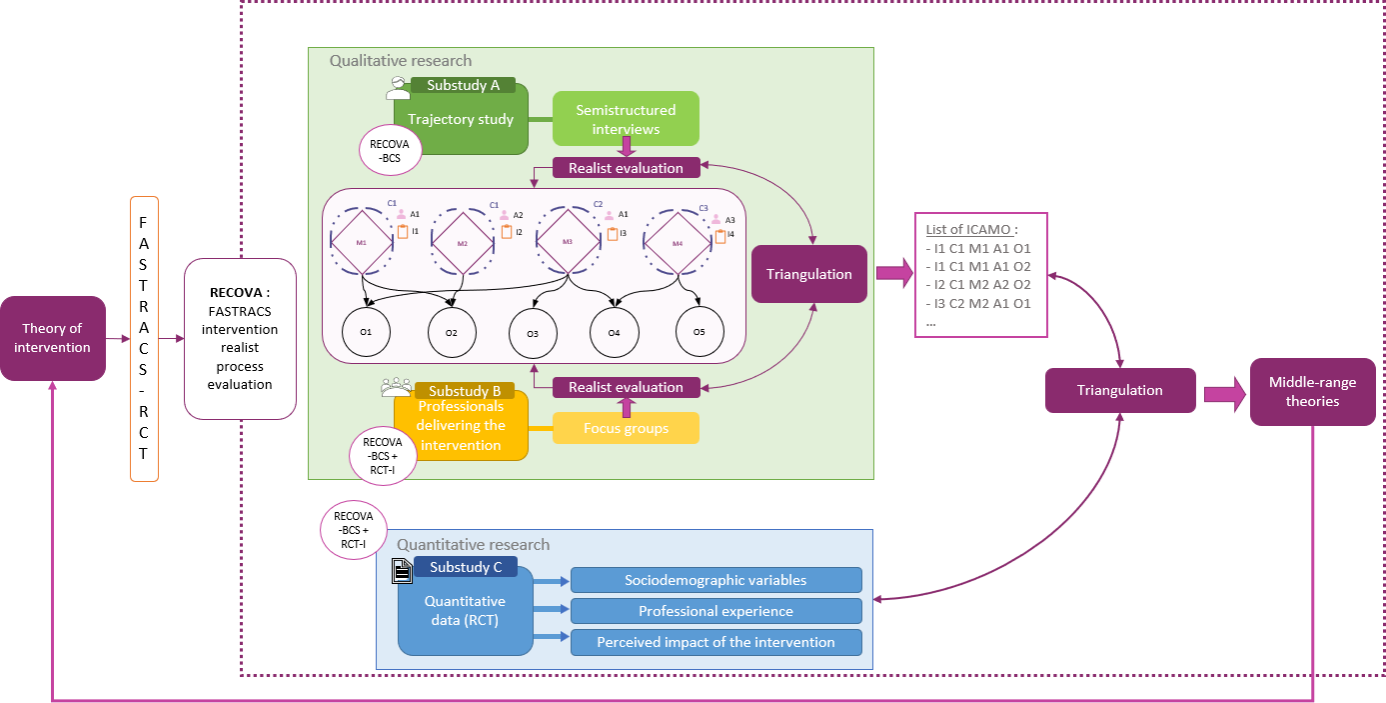
Evaluation of FASTRACS (FAcilitating and Sustain the Return to work After breast Cancer) intervention processes. BCS: breast cancer survivor; ICAMO: intervention-context-actor-mechanism-outcome; RCT: randomized controlled trial; RCT-I: BCSs who completed follow-up in the FASTRACS-RCT intervention group; RECOVA: Realist Collaborative Evaluation; RECOVA-BCS: sample of typical cases among RCT-I.

Figure 2 illustrates the realist evaluation of the FASTRACS intervention processes.

### Multidisciplinary Analysis and Stakeholder Engagement

The analysis will be led by an interdisciplinary team with expertise in general and occupational medicine, oncology, occupational health, social psychology, and public health. It will be enriched by the Intersectoral Strategic Participatory Committee, which includes 35 stakeholders (patients, associations, employers, professionals, and institutional representatives) involved throughout the project. The committee will contribute to interpreting findings and ensuring their relevance, feasibility, and potential for scale-up.

### Study Timeline

**Figure 3.**
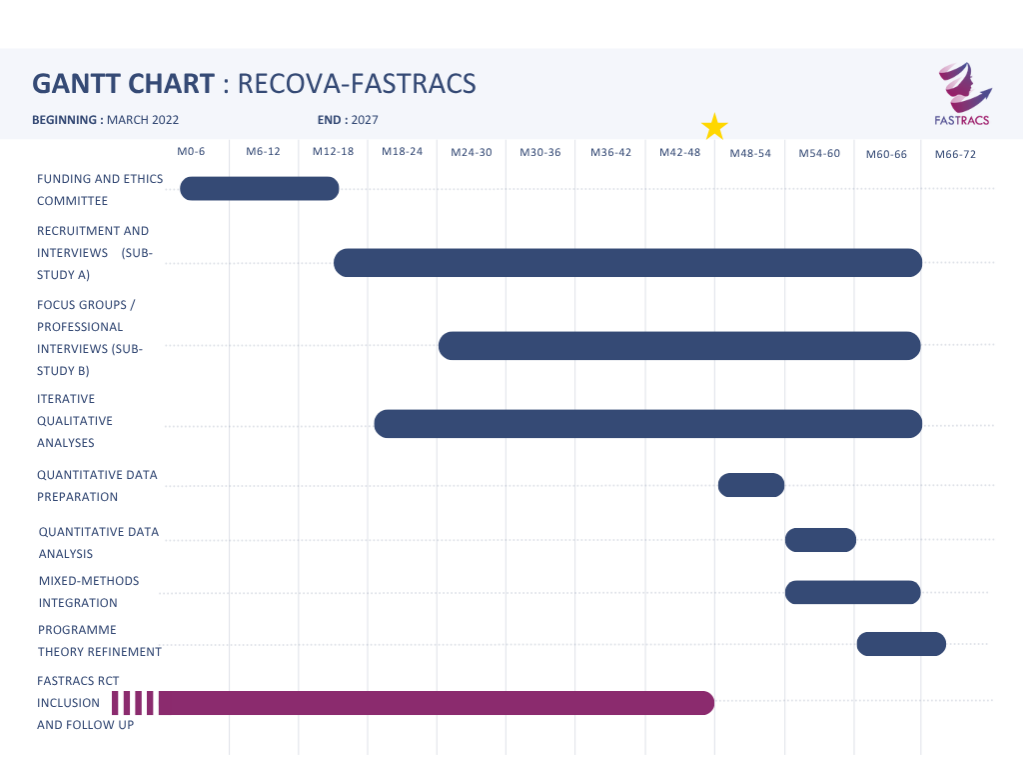
Timeline of the RECOVA-FASTRACS (Realist Collaborative Evaluation of a Work Disability Prevention Program for Breast Cancer Survivors) study. ★: end of FASTRACS randomized controlled trial follow-up.

A study timeline summarizing recruitment, qualitative and quantitative analyses, focus groups, mixed methods integration, and program theory refinement is presented in Figure 3.

### Ethical Considerations

This study was approved by the Advisory Committee on Information Processing in Health Research and by the French National Commission for Data Protection. Ethical approval for all participating sites was granted by the Research Ethics Committee of Lyon University Hospital (*Hospices Civils de Lyon*, approval 22‐5143). The FASTRACS-RCT received separate and dedicated ethical approval under the clinical trial regulatory framework [25].

Participants will receive verbal and written information outlining the study objectives, data confidentiality, and their right to withdraw at any time. In accordance with French regulations for noninterventional research, written consent is not required for participation in qualitative interviews. Oral informed consent will be obtained at several stages of the study. First, participants’ agreement to be contacted will be obtained by the clinical research associate involved in the FASTRACS-RCT. Approximately 1 week later, the researcher conducting the interview will contact participants again to confirm their willingness to participate and arrange the interview. At the beginning of each interview, study objectives, confidentiality procedures, and the voluntary nature of participation will be reiterated, and participants will be explicitly asked whether they still agree to participate before audio-recording begins.

RECOVA-BCS participants will provide written consent before any contact is made with individuals involved in their RTW trajectory (eg, health care professionals, workplace stakeholders, or relatives). No personal information disclosed by RECOVA-BCS participants will be shared with these individuals during interviews, and all data will be anonymized and securely stored in a collaborative workspace.

Given the potentially sensitive nature of discussions related to health status, work experiences, and RTW difficulties, particular attention will be paid to participants’ emotional well-being during interviews. The coordination team includes physician-researchers with clinical experience in mental health and supportive care. In the event of emotional distress or psychological difficulties identified during data collection, participants may be referred to appropriate care pathways or health care professionals, if needed.

Any substantial protocol amendment will be submitted for renewed ethics approval, and participant information materials will be updated accordingly.

## Results

The RECOVA-FASTRACS study received funding in 2022. Recruitment and qualitative data collection began in month 6.

Regarding substudy A, the initial sampling strategy, which planned to include approximately 20 RCT-I alongside up to 4 PTAs per participant (including general practitioners, occupational physicians, and workplace actors), has been achieved. Out of 15, 10 theoretical trajectories have been constituted. Analyses have been conducted iteratively.

Regarding substudy B, 1 out of 10 focus groups has been conducted. Data collection and analysis are expected to occur between months 54 and 60.

Regarding substudy C, the FASTRACS-RCT follow-up was completed in January 2026. Cleaning and preparation of quantitative implementation data are planned for the first half of 2026. Embedded integration and triangulation of qualitative and quantitative findings are expected to begin in month 54.

Final qualitative analyses are expected to be completed by the end of 2026. Dissemination of the main study findings is anticipated in late 2027.

## Discussion

The RECOVA-FASTRACS study is expected to provide a deeper understanding of how and under which conditions the FASTRACS intervention may support sustainable RTW after breast cancer. We hypothesize that this realist evaluation will help refine the initial FASTRACS program theory by identifying which components of the intervention appear to support or hinder RTW trajectories, for whom, and in which contexts. In particular, the study may contribute to the RTW literature by documenting the relevance of an ecological and multiactor approach involving health care professionals, occupational health teams, workplace stakeholders, and relatives around BCSs, in line with recommendations to take into account various aspects of the patient’s environment [48]. While current quantitative findings may help identify profiles of women for whom the intervention appears beneficial, the realist evaluation will further explore the underlying psychological mechanisms triggered by the intervention and the contextual conditions in which they are activated—mechanisms that are often only hypothesized in the existing literature.

The RECOVA-FASTRACS study uses a mixed methods approach, combining quantitative and qualitative data in a multiple-case study framework that enhances the internal and external validity of the results [47]. To our knowledge, this is the first application of realist evaluation to a program designed to support sustainable RTW after breast cancer. This theory-driven approach is well-suited to the complexity of RTW processes, which involve multiple stakeholders, diverse individual trajectories, and heterogeneous organizational contexts.

By identifying ICAMO configurations, the study will help decipher the causal pathways linking the intervention to its observed effects, clarifying what works, for whom, and under what conditions [30]. This realist evaluation design aligns with current methodological requirements for evaluating complex, multifactorial interventions, as recommended by the Medical Research Council [26,27]. Our study is also in accordance with public policy expectations, particularly those set out in France’s 10-year cancer strategy [15]. Furthermore, it offers the potential for transferability to other chronic diseases through the development of middle-range theories.

A major strength of this study is its embedded integration of qualitative and quantitative data at the individual level. This integration enables both within- and cross-case comparisons, allowing researchers to identify typical, deviant, and crucial cases [36]. This realist methodology is further enriched through regular consultations with stakeholders, including specialized local actors and patient advisers, ensuring that the results reflect the reality on the ground. Consequently, the study will better inform decision-making regarding the intervention’s subsequent scalability by stakeholders in other contexts.

Some limitations should, nevertheless, be acknowledged. As with all realist evaluations, findings will remain partly context-dependent, and some identified mechanisms may not be transferable to all organizational or health care settings. In addition, recruiting physicians throughout the study may prove challenging because of time constraints and workload, which could lead to uneven representation across professional groups [49].

A dissemination plan is also envisaged to ensure the timely transfer of findings to both scientific and operational stakeholders. Results from the implementation analysis are expected to be disseminated in 2027 through scientific publications and stakeholder-oriented outputs. In addition, the development of the FASTRACS middle-range theory, including tool-specific refinements, will be progressively disseminated between 2027 and 2028 as these theories are consolidated and refined across analyses.

## Supplementary material

10.2196/86608 Multimedia Appendix 1 Interview guide.

10.2196/86608 Multimedia Appendix 2 List of variables collected.

10.2196/86608 Checklist 1 GRAMMS checklist.

## Abbreviations

BCS: breast cancer survivor
CMO: context-mechanism-outcome
FASTRACS: Facilitating and Sustain the Return to Work After Breast Cancer
FASTRACS-RCT: Facilitating and Sustain the Return to Work After Breast Cancer–Randomized Controlled Trial
GRAMMS: Good Reporting of a Mixed Methods Study
ICAMO: intervention-context-actor-mechanism-outcome
PTA: program theory actor
RAMESES II: Realist and Meta-Narrative Evidence Syntheses—Evolving Standards
RCT: randomized controlled trial
RCT-I: Breast cancer survivors who completed follow-up in the FASTRACS-RCT intervention group
RECOVA-FASTRACS: Realist Collaborative Evaluation of a Work Disability Prevention Program for Breast Cancer Survivors
RTW: return to work
