# Supplementary material for: Realist Collaborative Evaluation of a Work Disability Prevention Program for Breast Cancer Survivors: Protocol for a RECOVA-FASTRACS Realist Evaluation
Source: JMIR Res Protoc. 2026 Jul 28;15:e86608. doi: 10.2196/86608 (PMC13411223; doi:10.2196/86608)
Supplement: Multimedia Appendix 1 [file resprot-v15-e86608-s001.pdf]

Interview guides

Interview guide - participant

Before the interview

When making contact, ask the participant to bring “traces” of the intervention (the tools) and, if necessary, bring the tools ourselves to help recall usage.

Introduction

Introduction of interviewer: My name is (name) and I am interviewer for the RECOVA study and I’ll be conducting this interview.

Presentation of the RECOVA study:
This interview is part of the research project on women’s return to work after breast cancer. This research fist studied what facilitates and what prevents women from returning to work after breast cancer.
Based on these data, the researchers created an intervention designed to facilitate this return to work, the FASTRACS study in which you took part. The intervention consisted of a Patient Guide, a consultation with your general practitioner (GP), a consultation with your occupational physician (OP) and a guide for your company.
We are now in the final phase of the research and looking to evaluate this intervention. Our aim is to gather your experience since the end of the end of your hospital treatment. In particular, we’re interested in your experience of the FASTRACS intervention. We want to explore your personal point of view and your journey, and won’t pass judgment on what you say.
We guarantee the confidentiality of our interview and the anonymity of your answers. The data collected will remain strictly within the scope of the research.
The interview will last about an hour. Interviews are usually recorded to facilitate transcription.
Do you agree to our exchanges being recorded? Do you have any questions before we start?

Questions

Opening questions:

How do you feel today in this first interview?

Can you introduce yourself in a few words?
If necessary, invite to give details about: family, professional situation

Have you returned to work, or is there a return date planned?

Theme: trajectory

Can you tell me how things have gone for you since you received the FASTRACS intervention’s tools?

What led you return to work?

What difficulties did you encounter in returning to work?

How were you supported in your efforts to return to work?

Were you helped by one or more people in particular (family, association, health professional, social worker, etc.)? If so, how?

Theme: work / cancer relationship

What does work mean to you?

What impact has your cancer had on your relationship with work?

How do you balance your work with your family life and personal activities?

What impact has your cancer had on your family/activities/work balance?

Theme: patient guide

Can you tell me about the moment when the patient guide was handed over? What do you remember about this moment?

Points to raise if not done spontaneously by the participant:
- differences between the experience of the moment at the time and the rereading of the experience afterward
- feelings about the explanations given at the time

How did you use the guide?

To help the participant remember how she used it, place the guide back in her daily life: where did she keep it at home? Is it with her medical file or elsewhere?
If not used: why not?

Points to raise if not done spontaneously by the participant:
- use of the guide with a third party (relative, doctor, etc.)
- use of other sources of information
- parts of the guide more or less used, for what reasons
- use of the guide to schedule a consultation with the GP

Would you say that this guide has helped you in your treatment? If yes/no why?

Dis this guide help you make decisions? If so which ones?

After exploring and explaining the participant's comments on this topic (or at the end of thee interview):
- ask the patient to take out her guide (annotations to be photographed with patient's agreement)
- take out your own guide if she doesn't have hers
- discuss the topic again with the help of the support

Theme: the general practitioner (GP)

What is your relationship with your GP?

How well does your GP know you? Do you feel at ease with him?

What do you remember about the consultation with your GP at the end of your radiotherapy / chemotherapy treatment?

How has your GP supported you since the end of your hospital treatment?
Do you think you were able to discuss all your needs with him/her and find answers?

Points to address if not done spontaneously by the participant:
- discussing the return to work with the attending physician
- collaboration with the OP and any other professionals (specialist, psychologist, physiotherapist, etc.)
- proposal for therapeutic part-time work
- opinion on frequency of follow-up by GP

Would you say that this consultation helped you in your treatment? If yes/no why?
Did this consultation helped you in your treatment? If yes/no why?

How was the memory aid used by your GP?
Was the use of this memory aid helpful? If yes/no why?

Theme: the occupational physician

What is your relationship with your OP?
How well does your OP know you? Do you feel at ease with him?

What do you remember about your pre-resumption / resumption visit with your OP?
How did your OP support you in your return to work?
Do you think you were able to discuss all your needs with him/her and find answers?

Points to address if not done spontaneously by the participant:
- collaboration with the attending physician
- contact with employer
- specific measures: job, accommodation, RQTH
- referral to other professionals?

Would you say that this consultation helped you in your career? If yes/no why?
Did this consultation help you make any decisions? If so, which ones?

How was the memory aid used by the OP?
Was it helpful to use this memory aid? If yes/no why?

Theme: company

What would you say about your relationship with your employer?

What was your relationship with your company while you were off work?
Can you tell me about your dealings with your company while you were off work?

Can you tell us your interactions with your company while you were off work?

What do you remember about getting back in touch with your company in preparation for the takeover?

Did you consult the employer’s guide? If so, what did you think of it?

How did you go about giving the guide to your employer, if you did?

To whom the guide has been given to?

If return to work: What do you remember about your return?

How did your colleagues and hierarchy welcome you?
Did you have the impression of experiencing unfair situations, and if so, which ones?
Did you feel that your needs (workload, work objectives, etc.) were heard?
Do you still have any questions or concerns about the new job? If so, what are they?

Points to address if not done spontaneously by the participant:
- *interview with a line manager*
- *meeting with colleagues*
- *organization of work (for you and your colleagues): renegotiation of work objectives?*
- *job adjustments?*

If job change: Why did you change jobs? How do you feel about this change?

If not returning to work: Where do you stand with regard to work today? What are your plans for the future?

Closing questions:

Overall, would you say that the FASTRACS intervention is useful for returning to work after breast cancer?

Is there anything else you'd like to add?

Thank you very much for your answers and participation. This interview is now over.

Interview guide - support person

Introduction

Introduction of interviewer: My name is (name) and I am interviewer for the RECOVA study and I'll be conducting this interview.

Presentation of the RECOVA study:
This interview is part of the research project on women's return to work after breast cancer. This research fist studied what facilitates and what prevents women from returning to work after breast cancer.
Based on these data, the researchers created an intervention to facilitate this return to work, the FASTRACS study, in which your *(link to participant)* took part. The intervention consisted of a Patient Guide, a consultation with the GP, a consultation with the OP and a Guide for the patient's company.
We are now in the final phase of the research and are looking to evaluate this intervention.
Mrs. X has designated you as the person who accompanied her on this post-cancer journey. Our aim is to gather your testimony and understand how you accompanied your *(link to participant)* during the phase following hospital treatment for breast cancer. No judgment will be passed on what you say.
We guarantee the confidentiality of our interview and the anonymity of your answers. The data collected will remain strictly within the scope of the research.
The interview will last about half an hour. Interviews are usually recorded to facilitate transcription. Do you agree to our exchanges being recorded? Do you have any questions before we start?

Questions

Opening questions:

Can you introduce yourself in a few words?
Age, family, situation, profession

<u>If the person works in a paramedical profession</u>:
What is your professional activity? Can you describe your daily routine?
If not mentioned spontaneously:
- length and location of practice
- type of patient base

Can you tell us about your relationship with your (*link with the participant*)?

<u>If the person works in a paramedical profession</u>:
How did Mrs. X find out about you? (Referral by a doctor, information given by an association…)

Theme: support

<u>If the person works in a paramedical profession</u>:
Can you tell me about your sessions with Mrs. X. during the time you were with her?
How did these sessions go?
In your opinion, what were the patient's main concerns during this period?
What do you think was at stake in these sessions?
Did these sessions evolve over the course of the treatment?

What facilitated these sessions (motivation, proximity to the practice, etc.)
What were the obstacles to carrying out these sessions (distance, pec time, cost, etc.)

If not mentioned spontaneously:
- number of sessions
- follow-up period

If the person works in a paramedical profession:
In your opinion, does Mrs. X. have any side effects of physical / psychological after-effects from her hospital treatments (radiotherapy, chemotherapy, surgery, hormone therapy…) and if so which ones?
If not mentioned spontaneously:
- impact on daily life
- impact on professional life

How did you support your (*link to participant*) during her time off work, and then when she returned to work if she did?
How did you think your (link to participant) experienced these different periods?

How do think you helped your (*link to participant*) in this process?
If not mentioned spontaneously: emotional support, administrative help, other…
If the support person is a paramedical professional:
- what advice did you give Mrs. X. about working after breast cancer?
- did you refer your patient to other healthcare professionals to manage these side effects and their repercussions? If so, which ones and why?

In your opinion, did other people (relatives, doctors, associations, others…) accompany your (*link with the participant*) on this journey? If so, in what way?

How did you experience your role as a support person for (*link to participant*) during the phase following hospital cancer treatment? (mental/emotional burden perceived…)
Did you encounter any difficulties in supporting (link to person) on work-related issues? If so, what are they?
Are you familiar with these issues?
If the support person is a paramedical professional: where do you find answers to your questions about these issues?

Theme: relationship to work

Can you tell me about your discussions with your (*link to participant*) about work issues?
In your opinion, how did your (link to participant) see her professional future?
In your opinion, what is your (link to participant)'s relationship with work?
How has this relationship changes over time?

What do you think led your (*link to participant*) to return to work?
What do you think was most helpful for your (link to participant)?

In your opinion, what difficulties did your (*link to participant*) encounter in returning to work?

Theme: use of tools

Has your (*link to participant*) used or discussed the patient guide with you, and if so, how?
Can you tell me about your discussions concerning this guide?

What did you think of the patient guide?

Closing questions:

In your opinion, has the FASTRACS intervention helped your (*link to participant*) to return to work?

Is there anything else you'd like to talk about or add?

Thank you very much for your answers and participation. This interview is now over.

Interview guide – General practitioner (GP)

Before the interview

When making contact, ask the GP to come with "traces" of the intervention (tools) and, if necessary, bring our own tools to help recall usage (memory aid, patient guide).

Introduction

Introduction of interviewer: My name is (name) and I am interviewer for the RECOVA study and I'll be conducting this interview.

Presentation of the RECOVA study:
This interview is part of the research project on women's return to work after breast cancer. This research fist studied what facilitates and what prevents women from returning to work after breast cancer.
Based on these data, the researchers created an intervention to facilitate this return to work, the FASTRACS study, in which your *(link to participant)* took part. The intervention consisted of a Patient Guide, a consultation with the GP, a consultation with the OP and a Guide for the patient's company.
We are now in the final phase of the research and are looking to evaluate this intervention.
Mrs. X has designated you as the person who accompanied her on this post-cancer journey. Our aim is to understand how your consultations with the patient went during the period following hospital treatment for breast cancer. In particular, we're interested in how you discussed professional issues with the patient. No judgment will be passed on what you say.
We guarantee the confidentiality of our interview and the anonymity of your answers. The data collected will remain strictly within the scope of the research.
The interview will last about an hour. Interviews are usually recorded to facilitate transcription. Do you agree to our exchanges being recorded? Do you have any questions before we start?

Questions

Opening questions:

Can you introduce yourself in a few words?
Age, type of general practice

Theme: trajectory

Can you tell me about your consultations with your patient during the period following hospital treatment for breast cancer?
What did you talk about during these consultations?
In your opinion, what were the patient's main concerns during this period?
What do you think was at stake during these consultations?

When and how did you discuss the question of work with your patient?
What place did work occupy among the subjects discussed with your patient?

In your opinion, what helped or motivated the patient to return to work?

In your opinion, what hindered or is still hindering your patient from returning to work? What difficulties were encountered?

How did you support your patient in her efforts to return to work?

What facilities or difficulties did you encounter?

What advice did you give your patient regarding work after breast cancer?

If not mentioned spontaneously:
- needs assessment
- proposal for a pre-takeover visit
- advice on maintaining contact with the company
- proposal for part-time work
- specific measures: disability, ...

Theme: recovery and after recovery

How would you describe your patient's recovery?

What were the positive aspects of this recovery? What difficulties did she encounter?

If not mentioned spontaneously:
- relations with employer, management
- relations with colleagues
- workstation adjustments
- changes in work organization

If new work interruption after return to work: for what reasons?

If change of position or no return to work: what are the reasons?

Theme: health/work relationship

Does your patient have any lasting side-effects or psychological/physical after-effects from her hospital treatments (radiotherapy, chemotherapy, surgery, hormone therapy, etc.) and if so, which ones?

In your opinion, what impact do the after-effects have on your patient's daily life?

In your opinion, what impact do these after-effects have on patient's professional life?

Have you referred your patient to other healthcare professionals to manage these side effects and their repercussions? If so which one and why?

If not mentioned spontaneously:
- physiotherapy?
- physical rehabilitation program?
- cognitive rehabilitation program?

- *surgery?*
- *psychology, psychiatrist, other?*

Theme: cancer/work relationship

What do you think motivated your patient to patient to work?

To what extent did cancer change her relationship with work?

Theme: role of family and friends

Did your patient talk to you about her relationships with her family and friends during the post-cancer period? If so, in what way?

Do you think the people around her supported her in her efforts to return to work? If so, in what way?

Theme: checklist

Your patient is supposed to have given you the checklist for the general practitioner that she received as part of the intervention: is this the case?
Show the checklist, if necessary, by giving a copy to the participant, which you can leave with her.

How did she introduce you to the checklist? At what point?

How did you use the checklist, if at all?
Did the checklist help you make any decisions? If so, which ones?
What differences did you notice between what you usually do in this type of consultation and what is suggested in the checklist?

What would you say about the checklist? Positive points / negative points?
What would you change in this checklist?

Theme: patient guide

Mrs. X has normally used a Patient Guide she received as part of the procedure.
Show the patient guide, if necessary, specifying that you should keep it for future interviews.
Did the patient tell you about the Patient Guide, and if so, how?

Did she use it with you?
What did you think of the patient guide?

How do you think it helped her? How did it help?

Theme: interprofessional coordination

What was your relationship with the patient's occupational physician (OP)?
Can you tell us about your exchanges with the patient's OP?
What do you think of your collaboration with the occupational physician in this particular situation and in general?

What other health professionals did you call on to support the patient during this period?
What do you think of these different collaborations?
What impact did these collaborations have on the patient's career path?

Closing questions:

Overall, how useful would you say the FASTRACS intervention was to Mrs. X in her return to work after breast cancer?

Is there anything else you'd like to add?

Thank you very much for your answers and participation. The interview is now closed.

Interview guide – Occupational health physician / occupational health nurse

Before the interview

When making contact, ask the GP to come with "traces" of the intervention (tools) and, if necessary, bring our own tools to help recall usage (memory aid, patient guide).

Introduction

Introduction of interviewer: My name is (name) and I am interviewer for the RECOVA study and I'll be conducting this interview.

Presentation of the RECOVA study:
This interview is part of the research project on women's return to work after breast cancer. This research fist studied what facilitates and what prevents women from returning to work after breast cancer.
Based on these data, the researchers created an intervention to facilitate this return to work, the FASTRACS study, in which your *(link to participant)* took part. The intervention consisted of a Patient Guide, a consultation with the GP, a consultation with the OP and a Guide for the patient's company.
We are now in the final phase of the research and looking to evaluate this intervention. Our aim is to gather your experience as an occupational physician/nurse in providing support when returning to work. In particular, we are interested in your use of the FASTRACS intervention tools, and the factors that may have contributed to facilitating or hindering her return to work. No judgment will be passed on what you say.
We guarantee the confidentiality of our interview and the anonymity of your answers. The data collected will remain strictly within the scope of the research.
The interview will last about an hour. Interviews are usually recorded to facilitate transcription. Do you agree to our exchanges being recorded? Do you have any questions before we start?

Questions

Opening questions:

Can you introduce yourself in a few words?

How long have you been practicing occupational medicine?

How long have you worked in this occupational health and prevention service?

How long have you known Mrs. X?

How long have you known the company where she works?

Theme: company / workstation / work team

What you say about this company? In terms of activity, work organization, number of employees…
If not mentioned spontaneously:
- *relations within the company? Social climate?*

What would you say about Mrs. X workstation? What constraints? What requirements?

What would you say about Mrs. X relations with her supervisor? And with her colleagues?

Theme: trajectory

Can you tell me how things have gone since you saw Mrs. X during her pre-reinstatement (or resumption visit if there was no pre-reinstatement visit)?

What has made it easier for her to return to work? In what way?
People:
- employer / supervisor
- colleagues
- ergonomist, psychologist, social worker
- GP, oncologist
- social security doctor

Measures:
- job adjustments
- resumption of part-time work
- interview with a line manager
- meeting with colleagues
- equipment
- training
- recognition as a disabled worker
- invalidity

On the contrary what could have prevented the return to work? What difficulties were encountered?

How were you able to support him in returning to work? What facilities or difficulties did you encounter?
Assessment of the employee's needs: how?
Recommendations for adapting the workstation: which ones, why?
Change of workstation: why, for what other position?

To what extend did you have the opportunity to discuss with the employer or supervisor? With other doctors or health professionals? *(Oncologist, surgeon, radiotherapist, social security doctor, other health professionals)*
With other resource people? *(Social worker, ergonomist, person in charge of job retention / job retention unit, other resource people)*

Theme: cancer/work relationship

What do you think motivated Mrs. X to return to work?

To what extent did cancer change her relationship with work?

Theme: the employer's guide

Mrs. X is supposed to have given you the Employer's Guide that she received as part of the treatment: is this the case?
Show the patient guide, if necessary, specifying that you should keep it for future interviews.

What was like to receive the Employer's Guide when she gave it to you?
- *at what point?*
- *what did Mrs. X say to you about the guide?*
- *did Mrs. X give you a second copy for the employer? Is so, who did she ask you to give it to?*

Would you say that this guide has helped you in your practice? If yes/no why?
Did this guide help you to make decisions? If so, which ones?

In concrete terms, how did you use the guide?
If not mentioned spontaneously:
- *reading: all at once, several times; easy/difficult to understand?*
- *parts more or less used/appreciated, for what reasons?*
- *transmission to the employer: when? how? to whom? why this person?*
- *if annotations by the OP: to be photographed, with his agreement*
- *to draw up the return-to-work plan / job retention strategy*

Theme: checklist

Mrs. X is supposed to have given you the checklist for the OP that she received as part of the intervention: is this the case?
Show the checklist, if necessary, by giving a copy to the participant, which you can leave with her.

How did she present the checklist to you? At what point?

What would you say about the checklist? Positive points/negative points?

How did the checklist help you? How did it help?
Did this checklist help you make any decisions? If so, which ones?

How did you use the checklist?
If not, why?
When, how, for what?

Theme: patient guide

Mrs. X normally used a Patient Guide that she received as part of the operation.
Show the patient guide, if necessary, specifying that you should keep it for future interviews.
Did she tell you about the Patient Guide?

Has she used it with you?

How do you think the Patient Guide helped her? How did it help?

Theme: Returning to work and afterwards

If returning to work: how would you say the return went?
In the first few days? In the weeks and months that followed?
Do you have any positive or difficult points to raise?
If not mentioned spontaneously:
- interview / relations with supervisors?
- meetings / relations with colleagues?
- job adjustments? Working hours?
- organization of work: renegotiation of work objectives?
- follow-up by the OP (medical check-ups, workstation study, discussion with the employer)
- follow-up by other resource people: which ones?

If change of workstation: why? Was the change voluntary or involuntary?

If new work stoppages after return to work: for what reasons?

If no return to work: why not?

Closing questions:

Overall, how useful would you say the FASTRACS intervention was to Mrs. X in her return to work after breast cancer?

Do you think that any particular tool (employer’s guide, checklist, patient’s guide) was particularly helpful?

Do you see anything that could be improved in the tools provided by the intervention?

Is there anything else you’d like to talk about or add?

Thank you very much for your answers and you participation. The interview is now over.

Interview guide – Company

Introduction

Introduction of interviewer: My name is (name) and I am interviewer for the RECOVA study and I'll be conducting this interview.

Presentation of the RECOVA study:
This interview is part of the research project on women's return to work after breast cancer. This research fist studied what facilitates and what prevents women from returning to work after breast cancer.
Based on these data, the researchers created an intervention to facilitate this return to work, the FASTRACS study, in which your *(link to participant)* took part. The intervention consisted of a Patient Guide, a consultation with the GP, a consultation with the OP and a Guide for the patient's company.
We are now in the final phase of the research and are looking to evaluate this intervention. Our aim is to gather your experiences since you received your Employer Guide. In particular, we're interested in what you experienced during this period and how you supported your colleague (*or collaborator*) as they returned to work at your company. No judgment will be passed on what you say.
We guarantee the confidentiality of our interview and the anonymity of your answers. The data collected will remain strictly within the scope of the research.
The interview will last about an hour. Interviews are usually recorded to facilitate transcription. Do you agree to our exchanges being recorded? Do you have any questions before we start?

Questions

Opening questions:

Can you introduce yourself in a few words and explain your relationship with the person returning to work?
Job title and seniority, nature of the relationship with the person, position / function of the person concerned

Could you briefly present your company?
If not mentioned spontaneously:
- type of company (public, private, non-profit)
- size
- sector of activity

Theme: RTW

How would you describe the patient's return to work?
What motivated her to return to work?
How did the whole team react to this return? (Discrimination or reluctance?)

How did you feel about the patient's return to work?

Theme: trajectory

How were you appointed as the main contact person for the person's reintegration into the company?

How the guide was given to you, when and by whom? Did other people also receive this employer's guide?

Did you use this guide?

If yes or no: why?
If yes in part: which tools in the guide in particular?
If yes: how did you use the guide?

Can you tell us how things have gone for you since you received the intervention tools?
If not mentioned spontaneously:
- detailed record of the stages in the trajectory
- breakdown of the work involved in supporting the takeover
- was the guide discussed (formally and/or informally)?
- how were decisions made?

Would you say that this guide has helped you provide you colleague with the best possible support to work?

What decisions has this guide helped you make?

Theme: Actions taken

How did you support the employee in her return to work?

Which type of action was taken? (adapting management, reorganizing work in the team, adjusting the workstation, working hours, discussions with the occupational physician, job study, reclassification, etc.)

Who (internally or externally) did you involve in making these adjustments? (ergonomist, OP, psychologist)?

Was it easy for you to set up this support? And using the guide?

When the person returns, have you taken any actions to help them reintegrate into the team and their work?
If not mentioned spontaneously:
- setting up a referral colleague/tutor
- sharing information with other people in the company
- meetings with the team, interview with the person

In your opinion, was the employee's return to work helped by support from her professional entourage to facilitate her reintegration?

Did you have recourse to other resources than the guide to help you in your support process?
If not mentioned spontaneously:
- looking for information
- advice from friends and family

Theme: Skills developed (if the participant is a manager)

Do you feel that you are better able to manage your employees' return to work following sick leave?

Would you say that the Employer's Guide has helped you to feel better informed about how to manage the absence and return to work of employees following illness?

Do you feel that you have had the necessary resources to maintain the skills of the person concerned so that they meet your company's performance needs?

Closing questions:

Overall, how useful would you say the FASTRACS intervention was to Mrs. X in her return to work after breast cancer?

Do you see anything that could be improved in the tools provided by the intervention?

Is there anything else you'd like to talk about or add?

Thank you very much for your answers and you participation. The interview is now over.
