# Supplementary material for: Realist Collaborative Evaluation of a Work Disability Prevention Program for Breast Cancer Survivors: Protocol for a RECOVA-FASTRACS Realist Evaluation
Source: JMIR Res Protoc. 2026 Jul 28;15:e86608. doi: 10.2196/86608 (PMC13411223; doi:10.2196/86608)
Supplement: Multimedia Appendix 2 [file resprot-v15-e86608-s002.pdf]

List of variables collected

Variable n°	Unit	Possible value 1	Possible value 2	Possible value 3	Possible value 4	Possible value 5	Possible value 6	Possible value 7	Possible value 8	Possible value 9	Possible value 10	Possible value 11	Possible value 12	Comments
1	Date of birth	Date	*NA*	*NA*	*NA*	*NA*	*NA*	*NA*	*NA*	*NA*	*NA*	*NA*	*NA*	*NA*
2	Age	Numeric	*NA*	*NA*	*NA*	*NA*	*NA*	*NA*	*NA*	*NA*	*NA*	*NA*	*NA*	*NA*
3	Occupation	Text	*NA*	*NA*	*NA*	*NA*	*NA*	*NA*	*NA*	*NA*	*NA*	*NA*	*NA*	*NA*
4	Link to the participant	List	Close	Professional										*Concerns only "trajectory" people*
5	Close links	Text	*NA*	*NA*	*NA*	*NA*	*NA*	*NA*	*NA*	*NA*	*NA*	*NA*	*NA*	*Concerns only "trajectory" people*
6	Professional relationship	Text	*NA*	*NA*	*NA*	*NA*	*NA*	*NA*	*NA*	*NA*	*NA*	*NA*	*NA*	*Concerns only "trajectory" people*
7	Trajectory person contacted	Yes / No	Yes	No										*Concerns only "trajectory" people*
8	Date of non-opposition to RECOVA FASTRACS	*NA*	*NA*	*NA*										
9	Date of the interview – focus group													
10	Interviewer conducting the interview													*Concerns only "trajectory" people*
11	Gender													*Concerns only "trajectory" people*
12	How long this person has known the participant													*Concerns only "trajectory" people*
13	Follow-up time													*Concerns only "trajectory" people*
14														
15														
16														
17														
18														
19														
20														
21														
22														
23														
24														
25														
26														
27														
28														
29														
30														
31														
32														
33														
34														

35														
36														
37														
38														
39														
40														
41														
42														
43														
44														
45														
46														
47														
48														
49														
50														
51														
52														
53														
54														
55														
56														
57														
58														
59														
60														
61														
62														
63														
64														
65														
66														
67														
68														
69														
70														
71														
72														
73														
74														
75														
76														
77														
78														
79														
80														
81														
82														
83														
84														
85														
86														
87														

88														
89														
90														
91														
92														
93														
94														
95														
96														
97														
98														
99														
100														
101														
102														
103														
104														
105														
106														
107														
108														
109														
110														
111														
112														
113														
114														
115														
116														
117														
118														
119														
120														
121														
122														
123														
124														
125														
126														
127														
128														
129														
130														
131														
132														
133														
134														
135														
136														
137														
138														
139														
140														

141														
142														
143														
144														
145														
146														
147														
148														
149														
150														
151														
152														
153														
154														
155														
156														
157														
158														
159														
160														
161														
162														
163														
164														
165														
166														
167														
168														
169														
170														
171														
172														
173														
174														
175														
176														
177														
178														
179														
180														
181														
182														
183														
184														
185														
186														
187														
188														
189														
190														
191														
192														
193														

194														
195														
196														
197														
198														
199														
200														
201														
202														
203														
204														
205														
206														
207														
208														
209														
210														
211														
212														
213														
214														
215														
216														
217														
218														
219														
220														
221														
222														
223														
224														
225														
226														
227														
228														
229														
230														
231														
232														
233														
234														
235														
236														
237														
238														
239														
240														
241														
242														
243														
244														
245														
246														

247														
248														
249														
250														
251														
252														
253														
254														
255														
256														
257														
258														
259														
260														
261														
262														
263														
263														
264														
265														
267														
268														
269														
270														
271														
272														
273														
274														
275														
276														
277														
278														
279														
280														
281														
282														
283														
284														
285														
286														
287														
288														
289														
290														
291														
292														
293														
294														
295														
296														
297														
298														
299														

300														
301														
302														
303														
304														
305														
306														
307														
308														
309														
310														
311														
312														
313														
314														
315														
316														
317														
318														
319														
320														
321														
322														
323														
324														
325														
326														
327														
328														
329														
330														
331														
332														
333														
334														
335														
336														
337														
338														
339														
340														
341														
342														
343														
344														
345														
346														
347														
348														
349														
350														
351														
352														

353														
354														
355														
356														
357														
358														
359														
360														
361														
362														
363														
364														
365														
366														
367														
368														
369														
370														
371														
372														
373														
374														
375														
376														
377														
378														
379														
380														
381														
382														
383														
384														
385														
386														
387														
388														
389														
390														
391														
392														
393														
394														
395														
396														
397														
398														
399														
400														
401														
402														
403														
404														
405														

406														
407														
408														
409														
410														
411														
412														
413														
414														
415														
416														
417														
418														
419														
420														
421														
422														
423														
424														
425														
426														
427														
428														
429														
430														
431														
432														
433														
434														
435														
436														
437														
438														
439														
440														
441														
442														
443														
444														
445														
446														
447														
448														
449														
450														
451														
452														
453														
454														
455														
456														
457														
458														

459														
