# Supplementary material for: Realist Collaborative Evaluation of a Work Disability Prevention Program for Breast Cancer Survivors: Protocol for a RECOVA-FASTRACS Realist Evaluation
Source: JMIR Res Protoc. 2026 Jul 28;15:e86608. doi: 10.2196/86608 (PMC13411223; doi:10.2196/86608)
Supplement: Checklist 1 [file resprot-v15-e86608-s003.pdf]

Title: REalist COllaborative eVAluation of a work disability prevention programme for breast cancer survivors: Protocol of the RECOVA-FASTRACS realist evaluation

RAMESES II reporting standards for realist evaluations: checklist

TITLE			Reported in document Y/N/Unclear/ Not applicable	Page(s) in document	Comment
1		In the title, identify the document as a realist evaluation	Y	1	
**SUMMARY OR ABSTRACT**					
2		Journal articles will usually require an abstract, while reports and other forms of publication will usually benefit from a short summary. The abstract or summary should include brief details on: the policy, programme or initiative under evaluation; programme setting; purpose of the evaluation; evaluation question(s) and/or objective(s); evaluation	Y	2	Abstract presented in study protocol format

TITLE			Reported in document Y/N/Unclear/ Not applicable	Page(s) in document	Comment
		strategy; data collection, documentation and analysis methods; key findings and conclusions<br><br>Where journals require it and the nature of the study is appropriate, brief details of respondents to the evaluation and recruitment and sampling processes may also be included<br><br>Sufficient detail should be provided to identify that a realist approach was used and that realist programme theory was developed and/or refined			

TITLE			Reported in document Y/N/Unclear/ Not applicable	Page(s) in document	Comment
**INTRODUCTION**					
3	Rationale for evaluation	Explain the purpose of the evaluation and the implications for its focus and design	Y	4-5	
4	Programme theory	Describe the initial programme theory (or theories) that underpin the programme, policy or initiative	Y	4	We described the intervention in the introduction and referred several times in the text to the forthcoming publication on the original programme theory
5	Evaluation questions, objectives and focus	State the evaluation question(s) and specify the objectives for the evaluation. Describe whether and how the programme theory was used to define the scope and focus of the evaluation	Y	5-6	
6	Ethical approval	State whether the realist evaluation required and has gained ethical approval from the relevant authorities, providing details as	Y	15-16	

TITLE			Reported in document Y/N/Unclear/ Not applicable	Page(s) in document	Comment
		appropriate. If ethical approval was deemed unnecessary, explain why			
**METHODS**					
7	Rationale for using realist evaluation	Explain why a realist evaluation approach was chosen and (if relevant) adapted	Y	5	
8	Environment surrounding the evaluation	Describe the environment in which the evaluation took place	Y	4	
9	Describe the programme policy, initiative or product evaluated	Provide relevant details on the programme, policy or initiative evaluated	Y	4-6	
10	Describe and justify the evaluation design	A description and justification of the evaluation design (i.e. the account of what was planned, done and why) should be included,	Y	5-15	

TITLE			Reported in document Y/N/Unclear/ Not applicable	Page(s) in document	Comment
		at least in summary form or as an appendix, in the document which presents the main findings. If this is not done, the omission should be justified and a reference or link to the evaluation design given. It may also be useful to publish or make freely available (e.g. online on a website) any original evaluation design document or protocol, where they exist			
11	Data collection methods	Describe and justify the data collection methods – which ones were used, why and how they fed into developing, supporting, refuting or refining programme theory	Y	7-15	

TITLE			Reported in document Y/N/Unclear/ Not applicable	Page(s) in document	Comment
		Provide details of the steps taken to enhance the trustworthiness of data collection and documentation			
12	Recruitment process and sampling strategy	Describe how respondents to the evaluation were recruited or engaged and how the sample contributed to the development, support, refutation or refinement of programme theory	Y	7-8	
13	Data analysis	Describe in detail how data were analysed. This section should include information on the constructs that were identified, the process of analysis, how the programme theory was further developed, supported, refuted and refined, and (where relevant) how analysis changed as the evaluation unfolded	Y	12-14	As it is a study a protocol we outline our analysis plan

TITLE			Reported in document Y/N/Unclear/ Not applicable	Page(s) in document	Comment
**RESULTS**					
14	Details of participants	Report (if applicable) who took part in the evaluation, the details of the data they provided and how the data was used to develop, support, refute or refine programme theory	Not applicable		Study protocol
15	Main findings	Present the key findings, linking them to contexts, mechanisms and outcome configurations. Show how they were used to further develop, test or refine the programme theory	Not applicable		Study protocol
**DISCUSSION**					
16	Summary of findings	Summarise the main findings with attention to the evaluation questions, purpose of the evaluation, programme theory and intended audience	Not applicable		Study protocol

TITLE			Reported in document Y/N/Unclear/ Not applicable	Page(s) in document	Comment
17	Strengths, limitations and future directions	Discuss both the strengths of the evaluation and its limitations. These should include (but need not be limited to): (1) consideration of all the steps in the evaluation processes; and (2) comment on the adequacy, trustworthiness and value of the explanatory insights which emerged<br><br>In many evaluations, there will be an expectation to provide guidance on future directions for the programme, policy or initiative, its implementation and/or design. The particular implications arising from the realist nature of the findings should be reflected in these discussions	Not applicable		Study protocol

TITLE			Reported in document Y/N/Unclear/ Not applicable	Page(s) in document	Comment
18	Comparison with existing literature	Where appropriate, compare and contrast the evaluation's findings with the existing literature on similar programmes, policies or initiatives	Not applicable		Study protocol
19	Conclusion and recommendations	List the main conclusions that are justified by the analyses of the data. If appropriate, offer recommendations consistent with a realist approach	Not applicable		Study protocol
20	Funding and conflict of interest	State the funding source (if any) for the evaluation, the role played by the funder (if any) and any conflicts of interests of the evaluators	Y	18-19	

Adapted from :
Wong G, Westhorp G, Manzano A, *et al*. RAMESES II reporting standards for realist evaluations. *BMC Med* 2016;14:96.

Good Reporting of a Mixed Methods Study (GRAMMS) checklist

GRAMMS Guideline Item	Section - page
1) Describes the justification for using a mixed methods approach to the research question	Method<br>Under “study design” page 6
2) Describes the mixed methods design (ie, the purpose, priority, and sequence of methods)	Method<br>Under “study design” pages 5 to 7 and “study timeline” page 15
3) Describes each method in terms of its sampling, data collection, and analysis	Method<br>Under “methods” pages 5 to 14 and “study timeline” page 15
4) Describes the integration of the quantitative and qualitative components (ie, where the integration has occurred, how it has occurred, and who among the research team has participated in it)	Method<br>Under “Embedded and iterative integration” page 13
5) Describes any limitation of one method associated with the presence of the other method	Does not provide an explicit description of the limitation, but this limitation can still be inferred.<br>Pages 9 to 11 Under “data collection”
6) Describes any insights gained from mixing or integrating methods	Does not provide an explicit description of the limitation, but this limitation can still be inferred.<br>Pages 9 to 15 Under “data collection” and “data analysis”

Adapted from
O’cathain A, Murphy E, Nicholl J. The Quality of Mixed Methods Studies in Health Services Research. J Health Serv Res Policy 2008;13:92–8.
